# Organic farmers use of wild food plants and fungi in a hilly area in Styria (Austria)

**DOI:** 10.1186/1746-4269-6-17

**Published:** 2010-06-21

**Authors:** Christoph Schunko, Christian R Vogl

**Affiliations:** 1 Working Group: Knowledge Systems and Innovations, Division of Organic Farming, Department for Sustainable Agricultural Systems, University of Natural Resources and Applied Life Sciences (BOKU), Gregor-Mendel Straβe 33, 1180 Vienna, Austria

## Abstract

**Background:**

Changing lifestyles have recently caused a severe reduction of the gathering of wild food plants. Knowledge about wild food plants and the local environment becomes lost when plants are no longer gathered. In Central Europe popular scientific publications have tried to counter this trend. However, detailed and systematic scientific investigations in distinct regions are needed to understand and preserve wild food uses. This study aims to contribute to these investigations.

**Methods:**

Research was conducted in the hill country east of Graz, Styria, in Austria. Fifteen farmers, most using organic methods, were interviewed in two distinct field research periods between July and November 2008. Data gathering was realized through freelisting and subsequent semi-structured interviews. The *culinary use value *(CUV) was developed to quantify the culinary importance of plant species. *Hierarchical cluster analysis *was performed on gathering and use variables to identify culture-specific logical entities of plants. The study presented was conducted within the framework of the master's thesis about wild plant gathering of the first author. Solely data on gathered wild food species is presented here.

**Results:**

Thirty-nine wild food plant and mushroom species were identified as being gathered, whereas 11 species were mentioned by at least 40 percent of the respondents. Fruits and mushrooms are listed frequently, while wild leafy vegetables are gathered rarely. Wild foods are mainly eaten boiled, fried or raw. Three main clusters of wild gathered food species were identified: leaves (used in salads and soups), mushrooms (used in diverse ways) and fruits (eaten raw, with milk (products) or as a jam).

**Conclusions:**

Knowledge about gathering and use of some wild food species is common among farmers in the hill country east of Graz. However, most uses are known by few farmers only. The CUV facilitates the evaluation of the culinary importance of species and makes comparisons between regions and over time possible. The classification following gathering and use variables can be used to better understand how people classify the elements of their environment. The findings of this study add to discussions about food heritage, popularized by organizations like Slow Food, and bear significant potential for organic farmers.

## Background

In Europe fast changing lifestyles and especially lack of time have recently caused a severe reduction of gathering wild plants and mushrooms [1,2], which in turn results in a loss of local knowledge about wild foods and about the local environment. This loss is serious for several reasons: gathering and use of wild plants and mushrooms is part of the cultural history of a region [3]; wild food species are part of people's local identity and traditions [4]; dishes made of wild foods are often identified as functional foods (foods with medicinal properties) [4,5]; and wild foods can contribute to overcoming periods of food shortage [4].

The above reasons make the preservation of local knowledge of gathering and use of wild food plants and mushrooms crucial. Several popular scientific publications, which aim to contribute to the preservation of wild food uses, have been released in German speaking countries (e.g. [6,7]). However, these publications often lack information about the origin, actuality, geographical distribution or cultural significance of the identified uses and species. Since wild food knowledge is context specific, in the sense that very different wild food species are used in distinct regions and uses of one and the same species can differ widely from one region to another [8], this lack of information weighs heavily. Instead detailed and systematic scientific investigation is needed for understanding and preserving wild food uses in distinct regions. The aim of this research is to accomplish such detailed investigations. We aim to explore wild food uses of farmers in the hill country east of Graz, to identify the culinary most relevant species and to make out local classification schemes.

In Europe, scientific studies on wild foods have only recently increased and research has concentrated in the Mediterranean area, especially Spain (e.g. [2,9-11]), Italy (e.g. [1,12,13]), France and Greece [e.g. [8] for both] are countries in which multiple investigations were conducted.

In Central and Eastern Europe research on gathering and use of wild foods was rather limited recently. However, the difficult historic and political situation until the mid (Central Europe) or end (Eastern Europe) of the 20^th ^century allows us to assume that local knowledge about wild food plants has been and may still be prevalent in several areas [3]. Historic and recent sources for e.g. Poland [14], Hungary [15,16], Bosnia-Herzegovina [17], Slovenia [18] or Eastern Europe [19] acknowledge this.

For the research area at hand, no previous systematic studies on wild food uses could be elicited, although indications for plant and mushroom gathering were found: the anthropologist Gamerith, who did extensive research on styrian peasant food in the mid-20^th ^century, wrote that "myriads of fruits and herbs gathered from nature and homegardens enriched the table" of peasants "and were snacked between the meals" [20]; an ethnographic article about peasant food in a valley in southwestern Styria mentions several wild gathered plant species used for salads (*Taraxacum sp*., *Cichorium sp*., *Nasturtium sp*., *Hieracium sp*., *Crocus vernus*) and for omelets (*Urtica sp*., *Achillea sp.*, *Glechoma hederacea*) as well as fungi gathered for food (*Boletus edulis*, *Clavaria aurea*, *Tricholoma gambosum*, *Tricholoma portentosum*, *Tricholoma terreum*, *Tricholoma equestre*, *Sparassis crispa*, *Polyporus squamosus*, *Clitopilus prunulus*, *Agaricus arvensis*, *Lactarius volemus*, *Russula virescens *and other *Russula sp*.) [21]; the styrian dictionary "Steirischer Wortschatz", published in the year 1903, also lists wild foods and wild food uses [22]; and Ferk lists, in the year 1910, 189 styrian names for fungi and investigates the etymology of the herrenpilz (*Boletus edulis*), pfifferling (*Cantharellus cibarius*) and täubling (*Russula sp.*), obviously important mushrooms in the area [23]. Besides that, leaflets explaining and promoting the gathering of fruits, herbs, spices and fungi for own consumption and selling were published in the years 1916 [24] and 1942 [25]. These leaflets were released during the first and second world war, when food supplies were scarce, and the exploitation of all available food sources became necessary.

Moreover, research on wild gathered food species adds to the discussion about food heritage, popularized by organizations like Slow Food [26], since wild food uses are often traditional ones. Wild foods also have potential as innovative products in organic farming as, following the Council Regulation (EC) No 834/2007 on organic production, wild food species can be certified as organic.

## Methods

Research was conducted in the hill country east of Graz, Styria, in Austria. The hill country is situated in the east of the provincial capital of Graz and covers an area of 215 km^2^. In total 29,000 people live there [27]. The annual precipitation averages 851 millimeters [28] and the average annual temperature is 9°Celsius [29]. The landscape is characterized by extended hills, divided into different sections by the *Raab*, *Feistritz *and *Lafnitz *rivers. Mixed deciduous forests prevail, dominated by *Carpinus betulus*, *Quercus robur*, *Quercus petraea*, *Fagus sylvatica*, *Castanea sativa *and *Prunus avium *subsp. *avium *[30].

The society of this region, before the Second World War, was marked by a highly agrarian population, with many people working on small units of land. After the war the expansion of agricultural production was the prime goal and in the subsequent decades agricultural production was increasingly rationalized and specialized [31]. Broiler poultry and pig production, in particular, experienced an important upturn and an increase in the production of corn accompanied this expansion. Furthermore, large scale fruit-growing became widely established [32].

Research was conducted between July and November 2008 and consisted of two distinct field research periods. In the first period, 15 farmers were interviewed. The addresses of the farmers were obtained by *Snowball Sampling *[33]. Seven organic farmers, whose addresses were randomly selected from a list of organic farmers in the area, presented the starting point. Farmers were selected as respondents since they are often knowledgeable in the customs of a region, work in food production and food preparation, work in and with nature and often live a more traditional lifestyle. Furthermore, organic farmers were selected in particular since the marketing of wild food products may represent a special marketing opportunity for them.

The sample comprised 12 organic and 3 conventional farmers, ten women and five men between the age of 34 and 61 (arithmetic mean: 49,8 years). All respondents, except one, were born and grew up in the research area. Nine respondents worked full-time on the farm whereas six respondents were part-time farmers. All respondents sold at least part of their products directly to final consumers.

In the first field research period *freelisting*, followed up by semi-structured interviews, was accomplished [33,34]. The freelisting question was: "Bitte zählen sie auf was in der Natur wächst und hier in der Umgebung gesammelt wird"; (literal translation: "Please list what grows in nature and is gathered in the neighborhood"). More detailed questions were then posed to investigate which parts of the plants are gathered and how the plants and mushrooms are used. In this paper, only the gathered wild plant and mushroom species used for food are presented.

In the second field research period, ten of these farmers were interviewed more thoroughly about the 22 most frequently listed plant and mushroom species (24 items since the flowers and the berries of *Sambucus nigra *and the flowers and the leaves of *Taraxacum sp*. are used in very distinctive ways and are therefore regarded as separate in the analysis) that were used as food. We determined if the plants and mushrooms were actually gathered in the years of 2007 or 2008, if they were gathered only from the wild or from cultivation, where they were gathered, at what distance from the farm and at which time(s) of the year.

Respondents' answers were written on prepared questionnaires during the interviews and entered into an *MS Access *database [35] afterwards [36]. Additionally all interviews were recorded with a *Philips Voicetracer 7890*.

The freelist data and the gathering and use variables were analyzed by frequency and percentages. The *use value *(UV) of plants, first developed by Phillips and Gentry [37] and adjusted by Tardío and Pardo-de-Santayana [38], was adapted to the *culinary use value *(CUV) in this study. The UV "transforms the complex, multidimensional concept of 'importance' into standardized and comparable numerical scales and values" [39] and therefore expresses the cultural value of plant or mushroom species quantitatively. The calculation of the UV is based on the frequency and diversity of use. Hence, the UV of a species is high, when it is used by many respondents and in diverse ways, and the UV is low, when it is used by few respondents and only for few uses (study [37-39] for closer explanations). While the UV of a species is calculated through the frequency and diversity of uses in distinct use categories (e.g.: edible, medicine, construction,...), the CUV is calculated through the frequency and diversity of use in distinct categories of culinary preparation (boiled, fried, roasted/baked, raw, dried/condiment). Therefore the CUV is an index indicating the culinary importance of a species as considered in different preparation categories. Moreover, species were merged into groups according to gathered plant part or mushroom and CUVs were calculated for these general categories (as performed with food-categories before [40]).

The gather and use variables of the 24 most common wild gathered plants and mushrooms used as food were used in *Hierarchical Cluster Analysis *(HCA) applying Ward method [9]. HCA was conducted to identify culture-specific logical entities of plants and mushrooms and their usage profiles. For the HCA the characteristics of the 24 items were depicted through 31 binomial variables in a matrix (1 = true; 2 = false). The variables used were related to the frequency of listing, frequency of gathering, the gathered plant parts, dishes in which the species are used, location of gathering, distance of gathering from the farm and the time of the year when the species were gathered. For the frequencies of listing and the frequencies of gathering the percentage of respondents who listed or gathered a species was used (<33%, 33-66%, >66%). All other variables were considered as true, if at least two respondents listed a variable as true. HCA displays the similarities of the species or variables in dendrograms, where species or variables with similar parameter values are placed in common clusters. After the creation of the dendrograms we related the clusters of species to clusters of variables by comparing the clusters with the raw data. HCA was accomplished in *SPSS 15.0 *[41].

For convenience, in this paper fungi, although recognized as distinct, are sometimes listed together with plants.

The results of this study were returned to the informants via a letter including the internet address to download the final paper of the project.

## Results

### Wild food plants

The informants mentioned edible plants and mushrooms a total of 150 times (including double entries) referring to 39 different species (Table 1). Every informant listed between 0 and 19 wild food species (arithmetic mean: 10; standard deviation: 5.6).

**Table 1:** Aggregated freelist of wild food species gathered in the hill country east of Graz (n = 15)*

Rank	Item	Frequency	Percentage	Average Rank
1	*Cantharellus cibarius*	12	80	5
2	*Boletus edulis*	12	80	5,5
3	*Rubus subgenus Rubus spp.*	11	73	4,727
4	*Macrolepiota procera*	11	73	7
5	*Taraxacum sp.*	10	67	6,8
6	*Russula sp.*	9	60	7,333
7	*Fragaria vesca*	9	60	5,444
8	*Rubus idaeus*	8	53	5,375
9	*Urtica sp.*	8	53	5,625
10	*Vaccinium myrtillus*	7	47	6,857
11	*Castanea sativa*	6	40	9
12	*Sambucus nigra*	5	33	3,2
13	*Lactarius sect. Deliciosi*	4	27	10
14	*Bellis perennis*	4	27	6,5
15	*Juglans regia*	3	20	9
16	*Rumex sp.*	2	13	4,5
17	*Plantago lanceolata*	2	13	2,5
18	*Amanita rubescens*	2	13	11
19	*Allium ursinum*	2	13	10
20	*Prunus avium *subsp. *avium*	2	13	12,5
21	*Glechoma hederacea*	2	13	8,5
22	*Thymus sp.*	2	13	8,5
23	*Nasturtium sp.*	1	7	7
24	*Galium odoratum*	1	7	7
25	*Armoracia rusticana*	1	7	11
26	*Achillea sp.*	1	7	1
27	*Coprinus sp.*	1	7	14
28	*Stellaria media*	1	7	14
29	*Plantago major*	1	7	3
30	*Ramaria botrytis*	1	7	17
31	*Xerocomus badius*	1	7	18
32	*Lepidium sp.*	1	7	19
33	*Juniperus sp.*	1	7	9
34	*Prunus domestica *subsp. *Syriaca*	1	7	11
35	*Sorbus aucuparia*	1	7	13
36	*Viola sp.*	1	7	14
37	*Primula sp.*	1	7	15
38	*Agaricus sp.*	1	7	7
39	*Sparassis sp.*	1	7	13

*Coding of variables: Frequency: number of respondents listing the item; Percentage: percentage of respondents listing the item; Average Rank: average rank of the item in individual freelists;

The wild food species listed most frequently are chanterelle mushroom (*Cantharellus cibarius*), edible boletus mushroom (*Boletus edulis*), blackberry (*Rubus subgenus Rubus spp.*), parasol mushroom (*Macrolepiota procera*), wood strawberry (*Fragaria vesca*), flirt mushroom (*Russula sp.*), wild raspberry (*Rubus idaeus*), nettle (*Urtica dioica*), dandelion (*Taraxacum sp.*), blueberry (*Vaccinium myrtillus*) and sweet chestnut (*Castanea sativa*). These plant and mushroom species were listed by 80 to 40 percent of the respondents. Eleven other plants and mushrooms were listed by 13 to 33 percent of the respondents. Seventeen wild food species were listed only once.

The 39 species belong to 24 different plant and mushroom families. The family with the most species cited is *Rosaceae *(6 species), followed by *Brassicaceae *and *Asteraceae *(3 species each), then *Lamiaceae*, *Plantaginaceae*, *Boletaceae*, *Agaricaceae*, *Russulaceae *and *Ramariaceae *(2 species each). For 15 families only one species was listed.

The wild foods are gathered from herbaceous plants (18 species), followed by mushrooms (11 species), trees and shrubs (5 species each). The items frequently gathered include: leaves (12 species), fruits and the mushroom bodies (11 species each) and flowers (6 species) (Table 2). Additionally for *Urtica dioica *the seeds and shoots as well as the root in the case of horseradish (*Armoracia rusticana*) are gathered.

**Table 2:** Gathering and preparation of the 24 most frequently listed wild food species*

Species	Local names	Gathered part	Preparation	Freq of gathering	Cult	Habitat	Distance	Season
*Cantharellus cibarius*	Eierschwammerl, Recherl	fungus	egg, gul, sce, sou, ric	gFF	W	woo	gCC, gC	spr, sum
*Boletus edulis*	Steinpilz, Herrenpilz	fungus	sou, sce, egg, gul, ric, bre	gFF	W	woo	gCC, gC, gA	sum
*Rubus subgenus Rubus spp.*	Brombeer	fruit	raw, mar, mil	gF	C	woo, mea, edgwoo	gCC, gC	sum, fall
*Macrolepiota procera*	Parasol	fungus	bre, fri	gFF	W	woo	gCC, gC	sum
*Fragaria vesca*	Walderdbeer	fruit	raw, mar, mil	gF	W	woo, mea, edgwoo	gCC, gC	spr, sum
*Russula sp.*	Täubling	fungus	fri	gF	W	woo	gCC, gC	sum
*Rubus idaeus*	Himbeer	fruit	raw, mar, mil	gF	C	woo, mea, edgwoo	gCC, gC, gA	sum, fall
*Urtica dioica*	Brennnessel	leaf	spn, sal, sou	gF	W	mea	gCC	spr, sum,fall
*Taraxacum sp.*	Löwenzahn	leaf	sal	gF	W	mea	gCC	spr
*Vaccinium myrtillus*	Schwarzbeer, Heidelbeer	fruit	raw, mar, swe	gF	C	woo, edgwoo	gCC, gA	sum, fall
*Castanea sativa*	Kastanie, Maroni	fruit	fri, coo, swe	gF	C	woo, edgwoo	gCC, gC	fall
*Taraxacum sp.*	Löwenzahn	flower	hon	gRA	W	mea	gCC	spr, sum
*Bellis perennis*	Gänseblümchen	leaf, flower	sal	gRA	W	mea	gCC	spr, sum
*Lactarius sect. Deliciosi*	Milchling	fungus	fri	gRA	W	woo	gCC	sum
*Sambucus nigra*	Holunder, Holler	flower	bak	gRA	C	woo, mea, edgwoo	gCC, gC	spr, sum
*Sambucus nigra*	Holunder, Holler	fructus	hko	gRA	W	mea, edgwoo	gCC, gC	sum, fall
*Juglans regia*	Walnuss, Nuss	fructus	raw	gRA	C	mea	gCC, gC	sum, fall
*Plantago lanceolata*	Spitzwegerich	leaf	sou	gRA	W	mea	gCC	spr, sum
*Rumex sp.*	Sauerampfer	leaf	sal, sou	gRA	W	mea	gCC	spr
*Thymus sp.*	Quendel	flower, leaf	con	gRA	C	mea	gCC	sum
*Allium ursinum*	Bärlauch	leaf	sou, sce, con	gRA	C	mea	gCC	spr
*Prunus avium *subsp. *avium*	Vogelkirsche	fruit	raw	gRA	W	woo, mea, edgwoo	gCC, gC	spr, sum
*Glechoma hederacea*	Gundelrebe	leaf	egg, sou	gRA	W	mea	gCC	spr, sum,fall
*Amanita rubescens*	Perlpilz	fungus	bre, sce	gRA	W	woo	gC	sum

*n = 15 for the variables: Gathered part, Preparation; n = 10 for the variables: Freq of gathering, Cultivation, Habitat, Distance, Season; Coding of variables: Preparation: ways of preparation or use: sou = soup, sce = sauce, con = condiment, bre = breaded, fri = fried, sal = salad, raw = raw, egg = with eggs, ric = with rice, mil = with milk (products), mar = marmalade, coo: cooked, spi: spinach, hon: *löwenzahnhonig*, bak: *gebackene hollerblüten*, hko: *hollerkoch*; Freq of gathering: frequency of gathering: plant species gathered by gRA = < 33%, gF = 33%-66%, gFF = > 66% each of all informants; Cult: cultivation of plants: C = also cultivated, W = gathered from wild only; Habitat: woo = wood, mea = meadow, edgwoo = edge of the wood; Distance: distance from farm: plant species gathered in gCC = < 0,2 kilometers, gC = 0,2-5 kilometers, gA = > 5 kilometers distance; Season: time of the year: spr = spring, sum = summer, fall = fall;

Due to the very distinctive use of the flowers and the berries of elderberry (*Sambucus nigra*) and the flowers and the leaves of *Taraxacum sp.*, these different plant organs are considered as different wild food plants in the following analysis.

### Gathering of the 24 most common wild food species

The wild food species are generally gathered in close proximity to the farms of the respondents. Especially for the herbaceous plants - *Taraxacum sp.*, *Urtica dioica*, perennial daisy (*Bellis perennis*), ribwort (*Plantago lanceolata*), sorrel (*Rumex sp.*), thyme (*Thymus sp.*), wild garlic (*Allium ursinum*) and ground ivy (*Glechoma hederacea*) - respondents indicated that they never go further than 200 meters away to gather these plants (in total 98/145 mentions for the category "less than 200 meters"). All other plants and mushrooms (except blusher mushroom (*Amanita rubescens*)) are gathered within 200 meters as well, but at times the respondents may travel up to 5 kilometers from their farms to harvest them (42/145 mentions for the category "200 meters to 5 kilometers"). It's rare that respondents gather species from a far distance away from the farm, and only *Vaccinium myrtillus*, *Rubus idaeus *and *Boletus edulis *were gathered further away than 5 kilometers in 2007/08 (5/145 mentions for the category "more than 5 kilometers") (Table 2).

The wild food species are mainly gathered from meadows (58/123 mentions for "meadows") and all species except the mushrooms and *Vaccinium myrtillus *come from meadows. The mushrooms are gathered from the forest and so are the fruits from all the various shrubs (42/123 mentions for "forest"). A number of foods are also gathered at the edge of forests: fruits from all shrubs, *Fragaria vesca*, wild cherry (*Prunus avium *subsp. *avium*), sweet chestnut (*Castanea sativa*) as well as *Cantharellus cibarius *(25/123 mentions for "edge of the forest") (Table 2).

Most of the wild foods are gathered in summer (73/135 mentions for "summer harvesting"). All herbaceous plants and *Sambucus nigra *flowers are also gathered in spring (37/135 mentions for "spring harvesting"). *Castanea sativa *and walnuts (*Juglans regia*) are gathered in fall as are the fruits from all shrubs (25/135 mentions for "fall harvesting") (Table 2).

The respondents also cultivate several of the plants from which they gather edible parts in the wild. These plants include: all the listed shrubs, namely *Rubus subgenus Rubus spp*. (6 respondents gather this plant from the wild/7 from cultivation), *Vaccinium myrtillus *(2/5), *Rubus idaeus *(5/7), *Sambucus nigra *flowers (8/1); the trees *Juglans regia *(2/9) and *Castanea sativa *(6/4); and the herbaceous plants *Allium ursinum *(1/2) and *Thymus sp*. (1/1) (Table 2). The other 16 wild food species, which were listed at least twice in the freelists, are gathered only from the wild.

### Culinary use value and preparation of the 24 most common wild food species

Wild food species in the hill country east of Graz are most often boiled, fried and eaten raw. Mushrooms have the highest culinary use value (CUV), followed by fruits, leaves and flowers. Mushrooms are mainly fried and boiled. Fruits are eaten raw, boiled and sometimes roasted or used in cakes. The leaves are eaten raw, boiled and fried. The flowers are eaten raw and fried (Table 3).

**Table 3:** Culinary use value by gathered part and preparation category (n = 15)*

Gathered part	CUV	Boiled	Fried	Raw	Roasted/Baked	Dried/Condiment	Others
Mushroom	6,73	2,40	4,00	0,00	0,00	0,20	0,13
Fruit	6,47	2,47	0,00	3,20	0,80	0,00	0,00
Leaf	2,67	0,67	0,60	0,93	0,00	0,20	0,27
Flower	0,53	0,00	0,20	0,33	0,00	0,00	0,00

**Total**		5,53	4,80	4,47	0,80	0,40	0,40

*Coding of variables: CUV: Culinary use value; Boiled: cooked, prepared as a soup, *gulasch*, rice, marmalade, compote; Fried: fried in oil, prepared as a spinach, as a sauce, with eggs or breadened and fried; Raw: eaten raw, prepared as a salad, mixed with milk (products) or sugar, macerated in sugar; Roasted/baked: roasted or baked in oven, tarts and cakes; Dried/condiment: used as a condiment, most often dried first; Others: used in spreads, *laibchen*, on pizza or for garnishing;

The species with the highest CUV are *Boletus edulis *and *Cantharellus cibarius*. The most common way of preparation is boiling, followed by frying. Boletus is also sometimes dried (Table 4). These two mushroom species are eaten in diverse ways, namely in soups, as a sauce, fried with eggs, as a *gulasch *and with rice (Table 2). The other mushroom species have lower CUVs. *Macrolepiota procera *is mainly eaten breaded and fried. *Russula sp.*, *Lactarius sect. Deliciosi *and *Amanita rubescens *are mainly fried.

**Table 4:** Culinary use value by species and preparation category including total number of uses and different uses (n = 15)

Species - Latin names	Species - English names	CUV	Boiled	Fried	Raw	Roasted/baked	Dried/condiment	Others	Uses	Different uses
*Boletus edulis*	boletus mushroom	2,47	1,13	1,07	0	0	0,20	0,07	37	8
*Cantharellus cibarius*	chanterelle mushroom	2,33	1,20	1,07	0	0	0	0,07	35	8
*Rubus subgenus Rubus spp.*	blackberry	1,47	0,73	0	0,73	0	0	0	22	7
*Rubus idaeus*	raspberry	1,20	0,53	0	0,67	0	0	0	18	7
*Fragaria vesca*	wood strawberry	1,20	0,40	0	0,80	0	0	0	18	5
*Vaccinium myrtillus*	blueberry	1,07	0,33	0	0,60	0,13	0	0	16	9
*Urtica dioica*	nettle	0,93	0,20	0,47	0,20	0	0	0,07	14	5
*Macrolepiota procera*	parasol mushroom	0,87	0	0,87	0	0	0	0	13	2
*Castanea sativa*	sweet chestnut	0,87	0,27	0	0	0,60	0	0	13	5
*Russula sp.*	flirt mushroom	0,67	0,07	0,60	0	0	0	0	10	2
*Taraxacum sp*. leaves	dandelion leaves	0,53	0,07	0	0,47	0	0	0	8	2
*Allium ursinum*	wild garlic	0,33	0,07	0,07	0	0	0,07	0,13	5	5
*Taraxacum sp*. flowers	Dandelion flowers	0,33	0	0	0,33	0	0	0	5	1
*Bellis perennis*	perennial daisy	0,33	0,07	0	0,20	0	0	0,07	5	4
*Sambucus nigra *fruits	elderberry fruits	0,27	0,20	0	0,07	0	0	0	4	2
*Lactarius sect. Deliciosi*	lactarius mushroom	0,27	0	0,27	0	0	0	0	4	1
*Juglans regia*	walnut	0,27	0	0	0,20	0,07	0	0	4	2
*Sambucus nigra *flowers	elderberry flowers	0,20	0	0,20	0	0	0	0	3	1
*Glechoma hederacea*	ground ivy	0,13	0,07	0,07	0	0	0	0	2	2
*Amanita rubescens*	blusher mushroom	0,13	0	0,13	0	0	0	0	2	2
*Thymus sp.*	thyme	0,13	0	0	0	0	0,13	0	2	1
*Rumex sp.*	sorrel	0,13	0,07	0	0,07	0	0	0	2	2
*Plantago lanceolata*	ribwort	0,13	0,13	0	0	0	0	0	2	1
*Prunus avium *subsp. *avium*	wild cherry	0,13	0	0	0,13	0	0	0	2	1

**Total**			5,53	4,80	4,47	0,80	0,40	0,40	246	85

The fruits of *Rubus subgenus Rubus spp.*, *Rubus idaeus*, *Fragaria vesca *and *Vaccinium myrtillus *have high CUVs. They are eaten raw, mixed with milk or milk products (like yoghurt or curd) or processed into jam.

*Urtica dioica *and *Taraxacum sp*. leaves are the leafy wild food plants with the highest CUVs. *Urtica dioica *is fried (often prepared as spinach), boiled or eaten raw, while *Taraxacum sp*. leaves are almost only eaten raw (often mixed with potatoes in a salad called *Röhrlsalat*). The other herbaceous plant species are mainly eaten in salads (*Bellis perennis, Rumex sp.*) and soups (*Plantago lanceolata, Rumex sp., Allium ursinum, Glechoma hederacea*).

For some plants very special ways of preparation were reported. The flowers of *Sambucus nigra *are dipped in batter and then fried (*Gebackene Hollerblüten*). The flowers of *Taraxacum sp*. are cooked or macerated in sugar to produce syrup (*Löwenzahnhonig*), which is used like a honey. The fruits of *Sambucus nigra *are processed with apples (*Malus domestica*), prunes (*Prunus domestica *subsp. *domestica*) and sugar to make a kind of jam (*Holunderkoch*).

The highest number of uses was listed for *Boletus edulis *(total of 37 uses including double mentions), *Cantharellus cibarius *(35 uses) and *Rubus subgenus Rubus spp*. (22 uses). The highest number of different uses was listed for *Vaccinium myrtillus *(nine different uses), *Boletus edulis *and *Cantharellus cibarius *(eight different uses each) (Table 4).

### Classification of the 24 most commonly used wild food species

**Figure 1:**
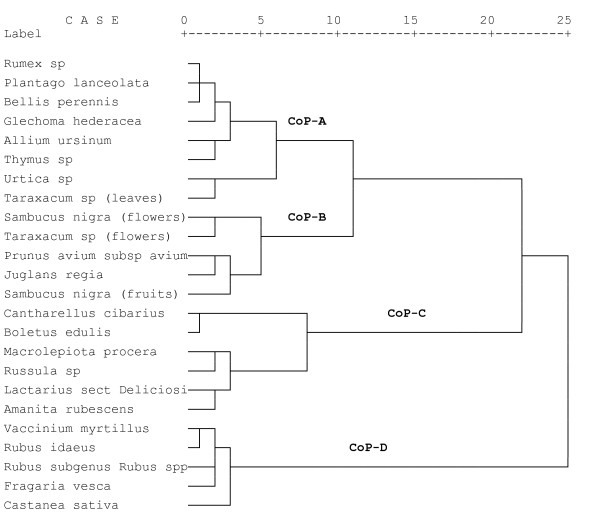
**Dendrogram of wild food species created through Hierarchical Cluster Analysis of gather and use variables (n = 10)**.

The classification of wild food species following HCA reveals four distinct clusters. These clusters consist of two times five, six and eight plants or mushrooms (Table 5, Figure 1).

**Table 5:** Description of cluster of species elicited through hierarchical cluster analysis (n = 10)

Cluster	Number of items	Overall Frequency	Std. Dev. (overall frequency)	Frequent Taxa	Label	Typical preparations	Description
CoP-A	8	4.6	3.4	*Taraxacum sp.*leaves; *Urtica dioica*	*kräuter*	*Löwenzahnsalat *(*Röhrlsalat*)*; Brennnesselspinat;*	Flowers and leaves, rarely listed and rarely gathered, used as condiment, in soups or salads, gathered in spring
CoP-B	5	5	2	*Taraxacum sp*. flowers; *Sambucus nigra *fruits and flowers	no label	*Löwenzahnhonig; Gebackene Holunderblüten; Holunderkoch;*	Fruits and flowers, gathered rarely, often used in unique preparations
CoP-C	6	8.3	4.3	*Cantharellus cibarius*; *Boletus edulis*;	*schwammerl*	*Schwammerlsuppe; Schwammersauce; Schwammerlgulasch;*	Mushrooms, very frequently listed and gathered, prepared with eggs, with rice, fried, as a sauce or breaded
CoP-D	5	8.8	2.4	*Rubus subgenus Rubus spp.*; *Fragaria vesca*; *Rubus idaeus*;	*beeren*	*Raw; Marmalade; Fruchtmilch; Fruchtjoghurt;*	Fruits, frequently listed and gathered, consumed raw, as jam or with milk (products), gathered from cultivated plants as well;

The HCA of gather and use variables also yields four clusters (Figure 2). The variables in the first cluster (CoV-1) are: "gathering of mushrooms", "very frequent listing" and "very frequent gathering" and the preparation of plants or mushrooms "with eggs", "with rice", "as a sauce", "fried" or "breaded". This cluster of variables matches with the CoP-C, containing all mushrooms. The CoP-C is divided into two subclusters at level 8. This division can be explained through the distinct ways of preparation since *Macrolepiota procera*, *Russula sp.*, *Lactarius sect. Deliciosi *and *Amanita rubescens *are consumed mainly fried or breaded and *Cantharellus cibarius *and *Boletus edulis *are rather prepared with eggs, with rice or as a sauce. The items of this cluster are labeled by the local term "*schwammerl*".

**Figure 2:**
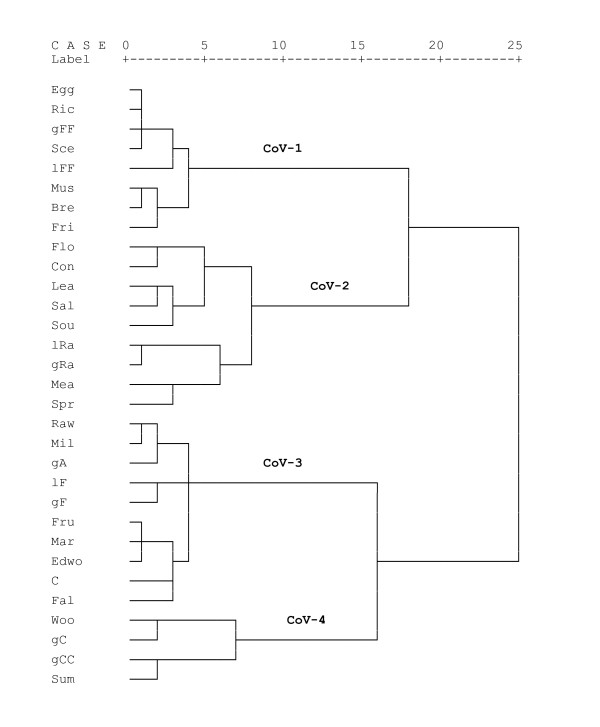
**Dendrogram of gather and use variables created through Hierarchical Cluster Analysis (n = 10)**. Coding of variables: Gathered part: Lea = Leaf, Flo = Flower, Fru = Fruit, Mus = Mushroom; Frequency of occurrence in the freelists: lRa = < 33%, lF = 33%-66%, lFF = > 66% each of all informants; Frequency of gathering: gRa = < 33%, gF = 33%-66%, gFF = > 66% each of all informants; Ways of preparation or use: Sou = soup, Sce = sauce, con = condiment, Bre = breaded, Fri = fried, Sal = salad, Raw = raw, Egg = with eggs, Ric = with rice, Mil = with milk (products), Mar = marmalade; Cultivation of plants: C = also cultivated; Habitat: Woo = wood, Mea = meadow, Edwo = edge of the wood; Distance from farm: gCC = < 0,2 kilometers, gC = 0,2-5 kilometers, gA = > 5 kilometers; Season: Spr = spring, Sum = summer, Fal = fall;

The second cluster of variables (CoV-2) consists of the variables: "use of the flowers", "use as a condiment", "use of the leaves", "preparation as a salad", "preparation as a soup", the "rare listing" and "rare gathering of the plant or mushroom", "gathering from meadows" and "gathering in spring". CoP-A matches with this cluster of variables. CoP-A is divided in two subclusters at level 6. This division can be explained since *Urtica dioica *and the leaves of *Taraxacum sp*. are gathered very frequently and not rarely like the other plants in this cluster. A further subcluster in the CoP-A occurs at level 3 and comprises *Allium ursinum *and *Thymus sp.*, which, in contrast to the other plants, also used as a condiment. The plants in this cluster are locally labeled "*kräuter*".

The third cluster of variables (CoV-3) incorporated the variables: "raw consumption", "consumption with milk or milk products", "consumption as jam", "gathering far away from the farm", "listed and gathered frequently", "gathering of fruits", "gathering from the edge of the forest", "gathering from cultivated plants as well" and "gathering in fall". This cluster matches with the CoP-D. Within the CoP-D, *Castanea sativa *represents a subcluster as this food is mainly roasted or cooked whereas the other plant foods are consumed raw or with milk or milk products. The items of this cluster (except *Castanea sativa*) are locally labeled "*beeren*".

The fourth cluster of variables (CoV-4) consists of the variables: "gathering in the forest", "gathering very close to the farm", "gathering close to the farm" and "gathering during summer". These four variables often occur together; however they are valid for multiple plants of several clusters. They cannot be clearly attributed to one cluster of plants and therefore comprise this distinct cluster.

The B cluster of plants (CoP-B) does not match very well with any of the clusters of variables. The plants in this cluster are gathered in meadows and are rarely gathered, which are variables of the CoV-2, but, contrary to CoV-2, they are not prepared in salads or soups but often in very unique ways. Also the fruits and flowers are gathered rather than the leaves. In the CoP-B the flowers of *Sambucus nigra *and the flowers of *Taraxacum sp*. set up a subcluster at level 5 since the flowers from both plants are gathered and in both cases they are prepared in unique ways (baked and as a "honey"). Due to the inconsistent composition of this cluster there is no local generic term that applies to it.

## Discussion

### Plant and mushroom species/habit/families

Among the eight most frequently listed plant and mushroom species, mushroom bodies (4 species) are gathered most often, then fruits (3 species) and leaves (1 species). Among the 14 most frequently listed species, fruits are listed most often (6 species), followed by mushroom bodies (4 species), leaves (2 species) and flowers (2 species). Following our study the gathering of mushrooms and fruits for food is therefore most common among farmers in the hill country east of Graz. However, our data is potentially biased since we collected data only during summer and autumn and not during spring, when most of the leafy vegetables and flowers are gathered.

The commonly gathered mushrooms and fruits are recorded as wild food species in many other areas as well [14,17,42-45]. Uses of leaves and flowers are known to only a few respondents. *Urtica dioica *and *Taraxacum sp*. leaves are the exceptions. Similar results were found in Poland, where the gathering of 15 species of fruits and only 2 of leafy vegetables is reported as common [14]. In other regions such as Spain [45], Bosnia-Herzegovina [17] or Italy [46] leafy vegetables were found as frequently gathered.

The best known and several of the less known edible mushroom species gathered in Styria today were in use at the beginning of the 20^th ^century already (*Boletus edulis, Cantharellus cibarius, Macrolepiota procera*, *Russula sp., Lactarius sect. Deliciosi, Agaricus sp., Sparassis sp.*) [21,23]. The use of *Taraxacum sp*. as salad, *Glechoma hederacea *in omelets and of *Urtica sp.*, *Achillea sp*. and *Nasturtium sp*. was also found in historic literature as well as in this study. However, the gathering of the mushrooms *Clavaria aurea, Tricholoma sp., Polyporus squamosus, Clitopilus prunulus *and the leafy vegetables *Cichorium sp*., *Hieracium sp*. and *Crocus vernus *was only encountered in historic literature [21].

The most important family in terms of wild food plants and mushrooms is *Rosaceae*. This is identical to findings in Poland [14] and similar to the Mediterranean area [47], where only species in *Asteraceae *are more important. Plants of *Asteraceae *have several, although rarely listed uses in the hill country east of Graz (one exception is the frequent use of *Taraxacum sp*. leaves for salad). This is similar to findings in Poland, where *Asteraceae *is the best represented family for green vegetables, although most of the uses are obsolete today. No plants of *Liliaceae *and *Apiaceae *were listed as gathered in the hill country east of Graz, although they are important wild food plant families in the Mediterranean area [47].

The field of ethnomycology is much younger than the field of ethnobotany [48] and wild gathered mushrooms are often neglected in ethnobiological studies still today [1]. However, the few ethnobiological studies which considered wild edible mushrooms in Europe found a diversity of gathered wild mushroom species. In Sicily 78 mushroom species are reported to be gathered and consumed [49] and in the southern Italian village of Castelmezzano 13 wild gathered mushroom species were registered [1]. In the hill country the gathering of wild edible mushrooms is also very common and some of the most salient wild food items are mushrooms. Eleven wild gathered mushroom species were recorded in this study. The use of *Boletus edulis*, *Cantharellus cibarius*, *Macrolepiota procera*, *Russula sp*. and *Lactarius sect. Deliciosi *is widespread and documented in many other areas as well [42,43,49].

### Culinary preparations

The gathered fruits in the Mediterranean are mainly consumed raw, as a jam or dried [47]. While eating fruits raw or as a jam is common in the hill country as well, the drying of fruits was not reported. Conversely the mixing of wild gathered fruits with milk or milk products, common in the hill country, was rarely found in other studies. However the mixing of *Vaccinium myrtillus *with milk or cream was documented for Poland [14].

Gathered greens in the Mediterranean are most often boiled and fried, stewed, eaten raw, and used in salads, omelets or pies [45,47]. In the hill country the boiling, frying and raw consumption of gathered leaves are known as well, whereas the preparation of omelets or pies with wild gathered leaves is almost absent. Only *Glechoma hederacea *was listed once to be used in omelets. The most widespread uses of gathered greens are for *Taraxacum sp*. leaves as a salad, often mixed with potatoes (*Solanum tuberosum*) (*Röhrlsalat*), and the preparation of *Urtica dioica *as a spinach.

The wild gathered mushrooms are most often roasted, stewed or eaten raw in southern Italy and Sicily [1,49], whereas in the hill country mushrooms are boiled or fried.

### Classification

In the hill country four different clusters of plant and mushroom species were found, as compared to Castilla-La Mancha, where eight clusters were elicited [9]. Three clusters are similar between the regions. In both areas leaves, used in soups and salads, mushrooms, which are fried, and fruits, used for making jam, are gathered.

Two different types of clusters, *species labeled *and *use labeled *ones, can occur, when plants or mushrooms are classified through usage patterns [9]. Species labeled clusters include *label species*, hence species which are used frequently by the informants, as well as rarely used species. The label species are widely known and used extensively while the other species in the cluster are often used as their substitutes. The standard deviation is high in species labeled clusters. In contrast, use labeled clusters do not have label species and a lower standard deviation [9]. In both kinds of clusters plant species are assembled because they are gathered and used in similar ways.

Following this concept, CoP-A and CoP-C are species labeled. Both clusters have label species (CoP-A: *Taraxacum sp*. leaves and *Urtica dioica; *CoP-C: *Cantharellus cibarius *and *Boletus edulis*), high standard deviation and several species that might substitute for the label species. Hence, for leaves and mushrooms the label species are used frequently, while the use of other species is less widespread. CoP-B and CoP-D are use labeled, since no label species occur and the standard deviation is low for both clusters (fruits and flowers in CoP-B are all rarely listed and the fruits in CoP-D are all frequently listed). Thus the species within these two clusters are used similarly often, and no outstanding, typical species can be named.

The classification following usage patterns does not follow the species concept and species boundaries and morphology of species are neglected [9]. This is true for CoP-B and CoP-D, where herbaceous plants, shrubs and trees are assembled in one cluster.

The clustering of wild food species following usage patterns represents a new way for discovering how people structure and manage their environment and cluster analysis helps to understand which species are selected from an environment and which are omitted [9]. However, it should be considered that the choice of variables used in cluster analysis may heavily influence the results. In this study eleven out of 31 variables were related to food preparation. Thus, special weight was given to food preparation and a different choice of variables might have yielded different results. Furthermore, it should be considered that this kind of classification is not fully emic, since researchers rather than the respondents select the variables to be used in the analyses.

## Conclusions

The gathering and use of several wild food species is common among farmers in the hill country east of Graz. Fruits and mushrooms in particular are gathered frequently. Wild leafy vegetables are gathered rarely, although a diversity of wild leafy food plants was documented. The plant and mushroom species gathered in the hill country are often the same as those gathered in other European regions. However, some uncommon preparations were found in the study area.

In past research culinary preparations of wild food species were presented most often without indicating the relative importance of the preparations (e.g. [1,9,11,49]). Sometimes the percentage of plant species prepared in special ways (e.g. [17]) or the frequency of citation of distinct uses is considered (e.g. [45]). However, a quantitative index indicating the culinary importance has been lacking. The culinary use value (CUV), as applied in this study, attributes culinary importance indices to both, distinct species and distinct ways of preparation. This facilitates the evaluation of the culinary importance of species and of the significance of distinct ways of preparation in the research area. Furthermore, this index makes the comparison of the relative importance of species and preparations between different regions, as well as over time, possible.

The concept of *"specialized" use value *(such as *"culinary" use value*) could be applied in other fields of study as well (e.g. as a *"medicinal use value" *for medicinally used plant species, or *"ornamental use value" *for plant species used for ornamental purposes). This further development of indices adds to quantification in ethnobotany.

The classification of wild food species through gathering and use variables reveals additional information about which wild food species are used in an area and about differences in preparation. This classification can be used to better understand how people classify their environment and how they select certain wild food species and neglect others. However, attention should be given to the selection of variables. In the hill country the classification shows that fungi, prepared in diverse ways, flowers and leaves used as spices, in soups or salads, and fruits, eaten raw, with milk (products) or as jam are the prevalent clusters of wild food uses.

This study found that local knowledge about wild food species and their uses is still existent among farmers in the hill country east of Graz. However, it can be supposed that important amounts of wild food knowledge got lost over the course of the 19^th ^and 20^th ^century through fundamental changes in the way of living in rural areas, which occurred everywhere in Europe [1,14,40]. The documentation of local knowledge is just a first step in order to reduce the loss of local knowledge. For its long-term survival, in-situ conservation, hence conservation by local people in place is essential [50]. Strategies for the local dissemination of local knowledge may include books, expositions, gardens and learning materials for schools [4]. Furthermore, organisations like Slow Food [26], which work in the area of food heritage and conservation of endangered food, can contribute to the revival of wild food uses [40]. This may result in an increasing demand for wild food species and finally will offer marketing possibilities for organic farmers, the informants of this study.

## Competing interests

The authors declare that they have no competing interests.

## Authors' contributions

CS carried out the study design, field research, analysis of data and writing of the paper. CRV substantially assisted in all stages of the study. Both authors read and approved the final manuscript.
